# TGF-β1-Induced Epithelial–Mesenchymal Transition Promotes Monocyte/Macrophage Properties in Breast Cancer Cells

**DOI:** 10.3389/fonc.2015.00003

**Published:** 2015-01-26

**Authors:** Joel Johansson, Vedrana Tabor, Anna Wikell, Sirpa Jalkanen, Jonas Fuxe

**Affiliations:** ^1^Department of Medical Biochemistry and Biophysics, Division of Vascular Biology, Karolinska Institute, Stockholm, Sweden; ^2^MediCity Research Laboratory, University of Turku, Turku, Finland

**Keywords:** epithelial–mesenchymal transition, breast cancer, immune cells, monocytes, macrophages, gene expression profiling, properties

## Abstract

Breast cancer progression toward metastatic disease is linked to re-activation of epithelial–mesenchymal transition (EMT), a latent developmental process. Breast cancer cells undergoing EMT lose epithelial characteristics and gain the capacity to invade the surrounding tissue and migrate away from the primary tumor. However, less is known about the possible role of EMT in providing cancer cells with properties that allow them to traffic to distant sites. Given the fact that pro-metastatic cancer cells share a unique capacity with immune cells to traffic in-and-out of blood and lymphatic vessels we hypothesized that tumor cells undergoing EMT may acquire properties of immune cells. To study this, we performed gene-profiling analysis of mouse mammary EpRas tumor cells that had been allowed to adopt an EMT program after long-term treatment with TGF-β1 for 2 weeks. As expected, EMT cells acquired traits of mesenchymal cell differentiation and migration. However, in addition, we found another cluster of induced genes, which was specifically enriched in monocyte-derived macrophages, mast cells, and myeloid dendritic cells, but less in other types of immune cells. Further studies revealed that this monocyte/macrophage gene cluster was enriched in human breast cancer cell lines displaying an EMT or a Basal B profile, and in human breast tumors with EMT and undifferentiated (ER−/PR−) characteristics. The results identify an EMT-induced monocyte/macrophage gene cluster, which may play a role in breast cancer cell dissemination and metastasis.

## Introduction

Metastasis is a complex multistep process whereby pro-metastatic cancer cells manage to break through cellular and molecular barriers and adapt to foreign microenvironments as they traffic to, and colonize distant organs (1, 2). The journey starts at primary tumor sites, where cancer cells invading through the basement membrane and into the surrounding tissue may migrate away from the primary tumor. For further trafficking and completion of the metastatic process, they need to intravasate into blood and lymphatic vessels, and stay alive in the circulation. Finally, they need to extravasate from vessels and propagate at distant sites. Based on both clinical observations and experimental data, it appears that only a minor fraction of cancer cells actually display all these pro-metastatic capabilities (1–4). Yet, it is not clear what these properties are, or how they are induced and maintained throughout the metastatic journey.

Epithelial–mesenchymal transition is considered to play an important role in the initial parts of the metastatic cascade – providing cancer cells with invasive, migratory, and cancer stem cell properties (5–7). Epithelial–mesenchymal transition (EMT) is a plastic process, and migratory cancer cells may cycle between epithelial and mesenchymal states (8, 9). Yet, less is known about the role of EMT in regulating trafficking of tumor cells in-and-out of blood and lymphatic vessels. The term “mesenchymal” is a rather unspecific definition. Although it may adequately describe the elongated, fibroblast-like morphology, and the invasive and migratory properties of EMT cells, it may not be sufficient to fully describe the properties of pro-metastatic EMT cells capable of disseminating through vessels and tissues. Clearly, fibroblasts and other type of normal mesenchymal cells do not migrate similar to metastatic cancer cells. Instead, the only cells that actually do traffic in our bodies similar to metastatic cancer cells are immune cells. In fact, immune cells are highly specialized in migrating through vessels and various tissue compartments as they migrate to different sites during inflammation.

Recently, it has been reported that circulating tumor cells (CTCs) in breast cancer patients may display epithelial, or mesenchymal/EMT properties, or a combination of both (10). Interestingly, the presence of CTCs with EMT properties was associated with worse prognosis. This suggests that migratory tumor cells that are capable of maintaining their EMT phenotype, not only within the primary tumor and its surroundings, but also as they migrate further and eventually reach the circulation, may represent a pool of pro-metastatic tumor cells.

We hypothesized that breast cancer cells not only being able to undergo EMT, but to sustain an EMT program, might develop properties that may be used at later steps of the metastatic process. In particular, we were interested to determine whether such EMT cells would acquire immune cell properties, which might be used for trafficking through the vasculature and dissemination to distant sites. To begin to address this, we performed gene expression profiling of mammary tumor EpRas cells that had been allowed to adopt an EMT program after long-term treatment with transforming growth factor beta 1 (TGF-β1) for 2 weeks. TGF-β1 is an inflammatory cytokine and a potent inducer of EMT (11). We found that long-term EMT cells indeed display properties of mesenchymal cells (fibroblasts). However, in addition, we identified a cluster of EMT-induced genes enriched in monocyte-derived immune cells including macrophages, mast cells, and myeloid dendritic cells (DCs). This cluster was enriched in human breast cancer cell lines classified as Basal B cells, and in human breast tumors negative for estrogen and progesterone receptors. It will be of interest for further studies to determine the role of genes within this cluster in breast cancer metastasis.

## Materials and Methods

### Cell culture

Mouse mammary EpRas tumor cells were a kind gift from the laboratory of H. Beug (Wien, Austria) and were generated through stable overexpression of the Ha-Ras oncogene in mouse mammary EpH4 epithelial cells (12). EpRas cells were cultured as adherent epithelial cells in DMEM/F12 medium supplemented with 10% fetal calf serum (FCS), 1% penicillin/streptomycin, and 1% l-glutamine. Cells were treated with 5 ng/ml recombinant human TGF-β1 (Peprotech Nordic, Stockholm, Sweden) for 48 h, or 2 ng/ml for 7 or 14 days (long-term). During treatment for 7 and 14 days cells were split every 3 days and new TGF-β1 was added in new DMEM/F12 medium supplemented with 10% FCS, 1% penicillin/streptomycin, and 1% l-glutamine. Cells were culture in a humidified incubator (37°C, 5% CO_2_). For gene expression analysis by microarray and q-PCR, 5 × 10^5^ cells of treated and non-treated cells were seeded in six-well plates 48 h prior to cells lysis.

### Immunofluorescence analysis

Cells grown on coverslips were fixed in absolute ethanol for 20 min. Samples were blocked in incubation buffer (5% normal goat serum and 0.1% BSA in PBS) for 1 h at room temperature. Primary antibody was diluted in 0.1% BSA/PBS and incubated 1 h at room temperature. The following primary antibodies and dilutions were used: mouse anti-E-cadherin (1:1000; clone 36, BD Biosciences, Stockholm, Sweden) and vimentin (1:1000; clone VI-10, Abcam, Cambridge, UK). Cells were washed five times in PBS/BSA and further incubated with secondary antibodies (1:1000 dilution) for 30 min and mounted in vectashield mounting media supplemented with DAPI (Vector Laboratories Ltd, Peterborough, UK). Immunoflouresent staining was assessed using a Nikon Eclipse microscope equipped for immunofluorescence.

### Gene expression analysis (Q-PCR)

Total RNA was extracted using an RNeasy mini kit (Qiagen, Valencia, CA, USA) according to manufacturer’s instructions. An amount of 2 μg of total RNA per sample was used for first-strand cDNA synthesis using the iScript cDNA synthesis kit (BioRad, Solna, Sweden). For q-PCR analysis, 5 ng of cDNA was used for PCR amplification by KAPA SYBR fast q-PCR kit (Kapa Biosystems, Wilmington, MA, USA) with validated QuantiTect primers (Qiagen). The following genes were analyzed: *Cdh1* (primer: QT00121163), *Ocln* (primer: QT00111055), *Cdh5* (primer: QT00110467), *Snai1* (primer: QT00240940), *Snai2* (primer: QT00098273), *Zeb1* (primer: QT00105385), *Zeb2* (primer: QT00148995), and *Twist1* (primer: QT00097223). The PCR was carried out as follows: 3 min at 95°C followed by 35 cycles of 3 s at 95°C, 20 s at 55°C, and 2 s extension step at 72°C in RotorGene RG-3000A PCR system.

### Western blot

Cells were lysed in RIPA buffer [50 mM Tris-HCl pH 7.5, 150 mM NaCl, 1 mM EDTA, 1% NP-40, 0.5% sodium deoxycholate, 0.1% SDS supplemented with protease inhibitors (Complete, Roche, Basel, Switzerland)]. Further, samples were boiled in Laemmli sample buffer (BioRad), separated by SDS–PAGE under reducing conditions, and transferred to a nitrocellulose membrane using the iBlot (Invitrogen, Carlsbad, CA, USA) dry transfer. The membrane was blocked using Blocking reagent (Roche) for 1 h and then incubated with primary antibodies over night at 4°C. Following antibodies were used: rabbit-α-LSP1 (1:500, HPA019693, Atlas, Stockholm), chicken-α Cotl1 (1:500) (13), rabbit-α-Dock8 (1:500, HPA003218, Atlas, Stockholm), and rabbit-α-Calnexin (1:1000) (14). Further secondary antibodies, α-mouse and α-rabbit Horseradish peroxidase IgG (1:8000, #7074, Cell Signaling Technology, Danvers, MA, USA), were incubated for 1 h at room temperature. All antibodies were diluted in blocking reagent (Roche). Immunoreactive bands were visualized by chemiluminescence (Roche) and developed using a LAS1000 system (Fuji Photo Film Co.).

### Microarray analysis

Microarray analysis was performed at the core facility for Bioinformatics and Expression Analysis (BEA) at the Karolinska Institutet. Total RNA was prepared from triplicate samples of EpRas cells that were either left untreated, or treated for long-term (14 days) with 2 ng/ml of TGFβ1 (Peprotech), and purified using RNeasy Mini Kit (Qiagen), supplemented with RNase-Free DNase (Qiagen). One hundred fifty nanogram of total RNA per sample was used to synthesize cDNA using a cDNA synthesis kit (Ambion/Life Technologies, Stockholm, Sweden). A GeneChip Sense Target Labeling kit (Affymetrix) was used to fragment and biotin label single stranded cDNA, which was probed on a MoGene-1_1-st-v1 array (Affymetrix) using an Affymterix Gene Titan instrument. Data were analyzed using the Robust Multi-array Average (RMA) algorithm. Gene expression data presented in this manuscript have been deposited to the Gene Expression Omnibus (GEO) database (Accession number: GSE59922).

### Cluster analysis of genes regulated during long-term induced EMT

Cluster analysis of genes upregulated or downregulated more than 1.5-fold was performed using the Database for Annotation, Visualization, and Integrated Discovery (DAVID)^1^, which is based on functional annotation of genes. Clusters with a *P* ≤ 0.05 were considered as significantly enriched.

### Meta-analysis of the expression of long-term induced EMT genes in immune cells

All 515 genes significantly upregulated by >1.5-fold in long-term EMT cells were analyzed for their degree of expression in fibroblasts (NIH3T3 and mouse embryonic fibroblasts) and different types of immune cells using the BioGPS database^2^. Gene expression was defined as “enriched” if the level in a certain cell type was higher than the average level for all 96 human tissues and cell lines included in the database. The following type of immune cells were included: Dendritic plasmacytoid (B220+), DCs lymphoid (CD8α+), DCs myeloid (CD8α−), Macrophage bone marrow, Macrophage (peri LPS thio 0 h), Mast cells, Granulocytes (Mac1+/Gr1+), NK cells, T cells FoxP3+, B cells GL7 positive Alum, B cells GL7 positive KLH, B cells (GL7 negative KLH), B cells (GL7 negative Alum), B cells marginal zone, T cells (CD4+), and T cells (CD8+).

One hundred twenty-three genes were found to be enriched in myeloid DCs, Macrophages, Mast cells, and Granulocytes, but not in fibroblasts, and were clustered into a monocyte/macrophage group, which was further analyzed.

### Meta-analysis of the expression of long-term induced EMT genes in human breast cancer cell lines

The 123 genes induced in long-term EMT cells and enriched in the monocyte/macrophage gene cluster were analyzed for their expression in datasets from 51 human breast cancer cell lines available from the European Bioinformatics Institute at the European Molecular Biology Laboratory (EMBL-EBI)^3^. The E-GEOD-41313 51 database, which contains microarray profiles (Affymetrix HT HG-U133+ PM Array Plate) of 51 human breast cancer cell lines, was used.

First, all tumor cell lines in the database were analyzed for the expression of EMT markers. The ratio between the average expression values of the mesenchymal markers *TWIST*, *SNAIL1*, and *VIM*, and the epithelial markers *OCLN*, *CLDN3*, and *CDH1*, was calculated for the 51 cell lines. Cell lines with a mesenchymal/epithelial ratio higher than 1.1 were defined as tumors displaying EMT characteristics. The average expression of the monocyte/macrophage gene cluster was compared between the EMT and non-EMT groups of cell lines. The expression value for each gene was normalized to the average expression of that gene in all samples. Statistical differences with *P* ≤ 0.05 were defined as significant.

Second, 38 of the human breast cancer cell lines were grouped into Luminal, Basal A, and Basal B cells, according to a previous classification (15). The average expression of the monocyte/macrophage gene cluster was compared between the different classes of cell lines and *P* ≤ 0.05 was considered as a significant difference.

### Meta-analysis of the expression of long-term induced EMT genes in human breast tumors

Genes within the monocyte/macrophage gene cluster were analyzed for their expression in a database (E-GEOD-36771) of microarray datasets (Affymetrix GeneChip Human Genome U133 Plus 2.0) from 107 human breast tumors (EMBL-EBI).

First, the average expression of genes in the monocyte/macrophage cluster was calculated for each tumor sample. Second, the average values were compared to ER/PR status, and the expression of EMT markers (using the same strategy as described for the tumor cell lines). *P* ≤ 0.05 was considered as a significant difference.

### Statistical analysis

Data represent means ± SEM with three independent experiments in triplicate. Statistical analyses were determined by using Student’s *t* test. *P* < 0.05 was considered as significant difference between the groups. In experiments with multiple groups, Bonferroni’s test for multiple comparisons was used, and *P* ≤ 0.05 was considered as significant.

## Results

### Long-term induction of EMT

**Figure 1:**
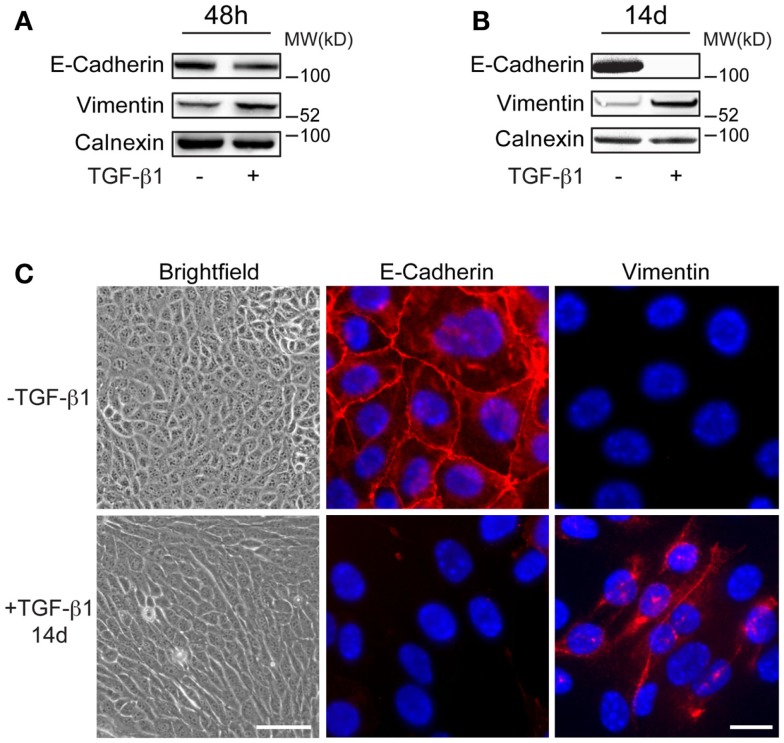
**Characteristics of long-term EMT cells**. **(A,B)** Western blot analysis showing more prominent changes in E-cadherin and vimentin expression in EpRas tumor cells after 14 days **(B)** compared to 48 h **(A)** of TGF-β1 exposure. Calnexin was used as a internal loading control. **(C)** Brightfield and immunofluorescence images showing that EpRas cells exposed to TGF-β1 for 14 days acquire characteristic features of EMT including an elongated, fibroblast-like morphology (left panels), loss of E-cadherin at cell–cell junctions (middle panels), and cytoplasmic staining of vimentin (right panels). Scale bars: 20 μm (left panels); 5 μm (middle and right panels).

Mouse mammary EpRas tumor cells were treated with 5 ng/ml of TGF-β1 for 48 h, or with 2 ng/ml for 7 days or 14 days (long-term) to study the effect on the commonly used EMT markers E-cadherin and vimentin. Immunoblotting analysis showed slight reduction in E-cadherin levels and increased expression of vimentin in EpRas cells that had been treated with TGF-β1 for 48 h, compared to non-treated cells (Figure 1A). After 7 and 14 days of TGF-β1 treatment, changes in E-cadherin and vimentin levels were more pronounced (Figure 1B; Figure S1 in Supplementary Material). After 14 days, E-cadherin was no longer detected in TGF-β1 treated cells. Microscopic analysis demonstrated that these cells had adopted an elongated, spindle-like shaped morphology, a characteristic feature of EMT (Figure 1C). Furthermore, immunofluorescence staining showed that E-cadherin was absent at cell–cell junctions while vimentin was localized in the cytoplasm in these long-term treated cells. Together, these results indicated that EpRas cells that had been treated with TGF-β1 for 48 h were in the induction phase of EMT and thus, displayed both epithelial and mesenchymal properties. In contrast, EpRas cells that had been treated with TGF-β1 for 14 days had lost epithelial features indicating that they had been reprogramed into mesenchymal-like cells. Based on these results, we decided to focus on profiling long-term EMT cells to identify their properties, based on gene expression.

### Long-term EMT cells display mesenchymal properties

**Figure 2:**
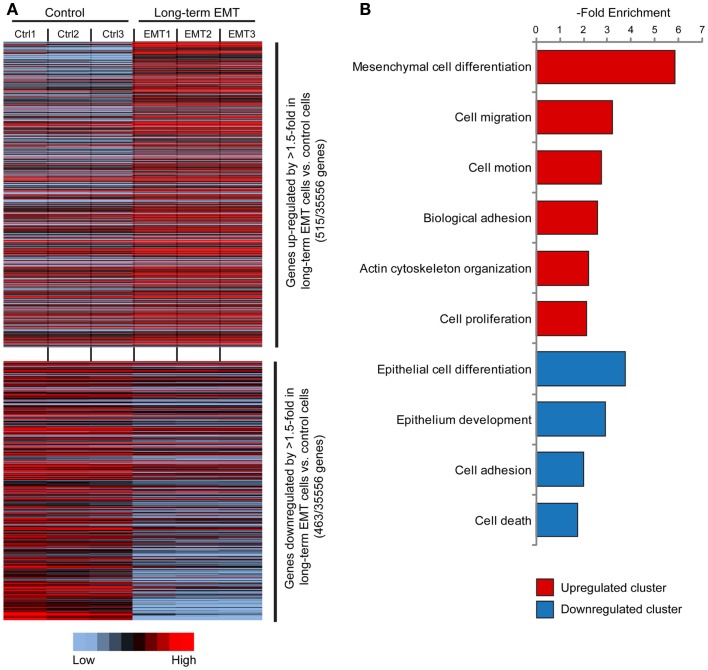
**Gene expression profiling of long-term EMT cells**. **(A)** Heat map showing genes significantly (*P* ≤ 0.05) upregulated (515 genes, upper part) or downregulated (463 genes, lower part) by >1.5-fold in long-term EMT vs. control EpRas cells. **(B)** Results from cluster analysis of genes significantly upregulated or downregulated in long-term EMT cells.

Microarray-based gene expression profiling of triplicate samples revealed that 515 genes were significantly upregulated, and 463 genes downregulated by more than 1.5-fold in long-term EMT cells compared to control cells (Figure 2A). The whole array set can be accessed at GEO database (Accession number: GSE59922). The 100 most upregulated and downregulated genes are displayed in Tables S1 and S2 in Supplementary Material). As expected, epithelial genes known to be inactivated during EMT in breast cancer cells, such as *Cdh1* (E-cadherin), *Ocln* (Occludin), *Epcam*, *Cldn7* (Claudin 7), *Id1*, *Krt7*, *Esr1*, *Cav2*, *Dsp*, *Gata3*, and *Cadm1* were among the downregulated genes. Reciprocally, known EMT-related genes, such as *Tnc*, *Mmp9*, *Jag1*, *Itgb3*, *Cdh5*, *Sema7a*, *Ctsw*, and *Itg5* were among the genes significantly upregulated in long-term EMT cells. Changes in the expression of several of these genes were validated by q-PCR (Figure S2A in Supplementary Material).

Interestingly, *Zeb1* and *Zeb2* were the only known EMT-inducing transcription factors that were upregulated in long-term EMT cells. In contrast, none of the master regulators of EMT – *Snai1*, *Snai2*, and *Twist1*, nor the EMT promoting factors *Tcf3/4*, *Lef1*, *Foxc2*, and *Ets1* were upregulated. In addition, the expression of known epigenetic regulators of EMT including *Hdac* factors (*Hdac1-6*) and *Mcm* factors (*Mcm 2-7*) was unchanged in long-term EMT cells. Expression of *Snai1*, *Snai2*, and *Twist1* instead appeared to peak at earlier time points as assessed by q-PCR analysis of EpRas cells treated for 48 h or 7 days with TGF-β1 (Figure S2B in Supplementary Material).

Cluster analysis showed that the induced genes were associated with mesenchymal cell differentiation, cell migration, and motility (Figure 2B; Table S3 in Supplementary Material). In addition, genes encoding proteins involved in non-epithelial cell–cell adhesion including various integrins (*Itga2*, *Itga5*, *Itga7*, and *Itgb3*) and cadherins (*Cdh5*, *Cdh10*, and *Cdh17*), as well as other cell adhesion molecules, such as *Ncam1*, *Alcam*, and *Mcam*, were induced. As expected, downregulated genes were linked to epithelial differentiation or development, and cell adhesion. Some downregulated genes were also associated with positive regulation of cell death.

### Identification of a monocyte/macrophage gene cluster induced in long-term EMT cells

**Figure 3:**
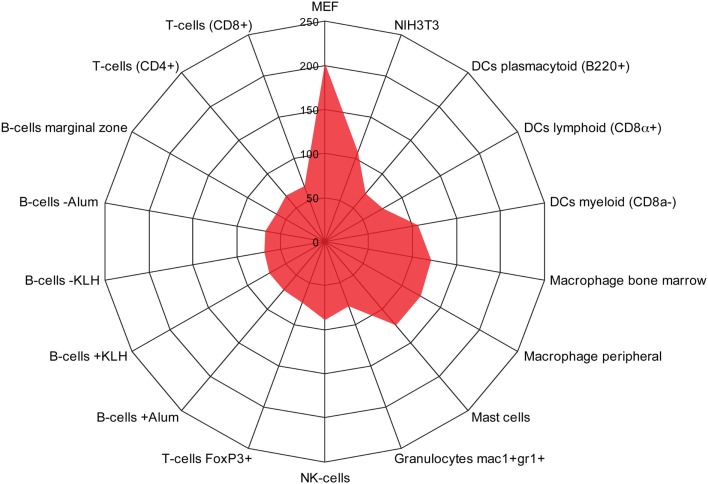
**Identification of a monocyte/macrophage cluster in long-term EMT cells**. Radar chart showing the number of genes upregulated in long-term EMT cells and enriched in mesenchymal cells [NIH3T3 and mouse embryonic fibroblasts (MEF)] or various types of immune cells. The highest number of genes was found in MEFs (202) followed by macrophages (bone marrow: 124, peripheral: 121), mast cells (124), NIH3T3 cells (108), and dendritic cells myeloid (106). Other immune cells had lower numbers that pended between 63 and 89. Thus, in addition to mesenchymal properties, properties of monocyte-derived cells were enriched in long-term EMT cells.

We used the BioGPS database to compare the expression of the 515 induced EMT genes in both mesenchymal cells (NIH3T3 and Mouse Embryonic Fibroblasts), and different types of immune cells. As expected, a large cluster of genes was enriched in fibroblasts (Figure 3). In addition, we found another cluster of 123 genes that was significantly enriched in monocyte-derived macrophages, mast cells, and DCs of a myeloid type, but not in B cells, T cells, other type of DCs, or granulocytes.

The largest pool of genes in this monocyte/macrophage cluster encodes plasma membrane proteins. Some of these, such as *Cd53*, *Gab2*, *Glipr1*, *Laptm5*, *Lsp1*, and *Rap2a* were enriched in different types of monocyte-derived cells (macrophages, mast cells, and myeloid DCs) (Table 1). Others, such as *Cnnm4*, *Kctd13*, *Fyn*, *Dag1a*, and *Sema6d* were more restrictively enriched in some of the cell types. A similar type of variation in enrichment between different genes and different type of immune cells was seen for the second and third largest sets of genes, which encode nuclear and cytosolic proteins, respectively. Western blot analysis was performed to validate changes in gene expression for one gene from each of the groups encoding plasma membrane (*Lsp1*), cytosol (*Dock8*), and cytoskeletal (*Cotl1*) proteins (Figure S3 in Supplementary Material).

**Table 1:** **List of genes induced by >1.5-fold in long-term EpRas EMT cells and enriched in human myeloid cells**.

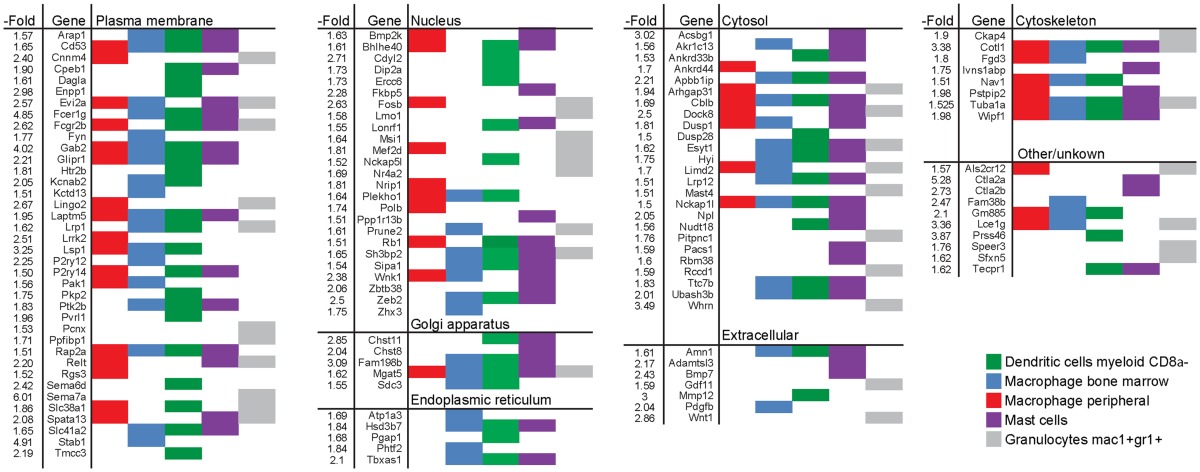

### Monocyte/macrophage gene cluster is linked to EMT in human breast cancer

**Figure 4:**
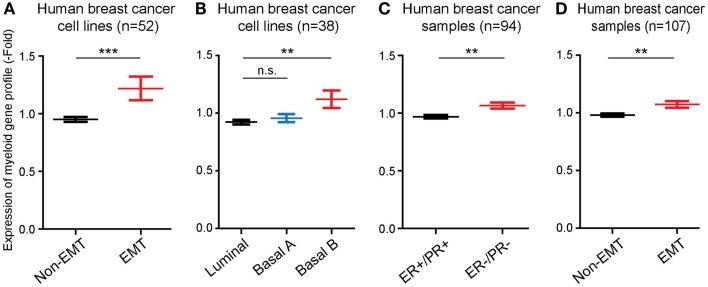
**The EMT-induced monocyte/macrophage cluster is linked to EMT in human breast cancer**. **(A,B)** Bar graphs showing that the average expression level of the EMT-induced monocyte/macrophage cluster was increased in human breast cancer cell lines displaying EMT vs. non-EMT properties, and in basal B cells compared to luminal and basal A cells. **(C,D)** Bar graphs showing that the average expression level of the EMT-induced monocyte/macrophage cluster was increased in human breast cancer samples negative for estrogen and progesterone receptors (ER−/PR−) compared to ER+/PR+ tumors, and in tumors displaying EMT vs. non-EMT properties.

Next, we performed studies to determine whether the monocyte/macrophage gene cluster, which we found was induced in long-term EMT cells, could be linked to EMT in human breast cancer cells and tumors. For this, we took advantage of published microarray-based datasets available through the EMBL-EBI database. First, we compared the expression levels of genes within the monocyte/macrophage cluster in 51 human breast cancer cell lines. We found that the average expression of the genes was significantly (*P* ≤ 0.001) higher in cell lines that displayed features of EMT, compared to non-EMT cell lines (Figure 4A). In addition, we compared the expression of the gene cluster in breast cancer cell lines previously classified as Basal B, Basal A, or Luminal cells (15). In these earlier studies, it was found while luminal breast cancer cells appeared most differentiated, Basal cells were less differentiated. In particular, Basal B cells were found to display EMT features. We found that the monocyte/macrophage gene cluster was significantly more expressed in cells classified in Basal B compared to Luminal or Basal A cell lines (*P* ≤ 0.01) (Figure 4B).

Finally, we also compared the expression of the EMT-induced monocyte/macrophage gene cluster in samples of human breast cancer. The gene cluster was significantly more expressed (*P* ≤ 0.01) in estrogen receptor and progesterone receptor negative tumors (ER−/PR−), which are classified as the most aggressive and least differentiated breast tumors (16), compared to ER+/PR+ tumors (Figure 4C), as well as in EMT tumors compared to non-EMT tumors (*P* ≤ 0.01) (Figure 4D).

## Discussion

We tested the hypothesis that breast cancer cells that are reprogramed through TGF-β1-induced EMT acquire properties of immune cells. To study this, we performed microarray-based gene profiling of tumor cells that had been allowed to adopt an EMT program for 14 days. In addition to the expected mesenchymal traits that were induced in EMT cells, we identified a gene cluster, which is enriched in monocyte-derived immune cells including macrophages, mast cells, and myeloid DCs.

Compared to most previous studies of gene expression changes during TGF-β1-induced EMT, which have focused on the first 24–72 h (17, 18), we decided to profile tumor cells that had been allowed to adopt an EMT phenotype for 14 days. There were several reasons behind this approach. First, since the *early phase* of TGF-β1-induced EMT is paralleled by growth inhibitory and apoptotic responses (19), we figured that many gene expression changes observed during this phase might not be primarily related to the EMT response. However, with time, growth inhibitory and apoptotic responses are released while EMT proceeds (20, 21). Thus, we figured that profiling the *late phase* of EMT might give valuable information about the transcriptional changes involved in reprograming tumor epithelial cells into mesenchymal cells. In comparison, reprograming of differentiated mouse and human cells into pluripotent stem cells takes weeks, rather than days (22, 23). Second, since recent results show that tumor cells maintaining an EMT profile in the circulation are the most capable ones in forming distant metastasis (10), we speculated that cells capable of maintaining EMT for a sustained period might display properties that could be important for later steps of the metastatic process including intra- and extravasation.

As expected, cluster analysis showed that known epithelial genes and cell adhesion molecules were the most downregulated in long-term EMT cells. In addition, genes encoding positive regulators of apoptosis were downregulated. We find this interesting since EMT has been linked to survival and resistance to apoptotic stimuli in breast cancer (24). In contrast, master regulators of EMT including *Snai1*, *Snai2*, and *Twist1* were not upregulated in long-term EMT cells, but peaked at earlier time points. Among known EMT inducers only *Zeb2*, which encodes Smad-interacting protein-1 (*Sip1*) (25, 26), was upregulated in long-term EMT cells. This was somewhat surprising given the fact that these EMT inducers are known to be rapidly induced and vital for the induction of EMT (7, 27–33). However, other results show that after the induction of EMT, the expression of EMT inducers, such as *Snai1*, declines to almost baseline levels (34).

Together with our data, this suggests that while *Snail1* and other EMT-inducing factors are critical for the early phase of EMT, other mechanisms come into play at later stages of EMT, as cells are reprogramed into mesenchymal cells. Such mechanisms are likely to play important roles in maintaining cells in an EMT state but might also provide EMT cells with properties used at later stages of the metastatic process. In line with this, we found that several of the most upregulated genes in long-term EMT cells including *Tnc*, *Mmp9*, *Jag1*, and *Ret*, are known to promote breast cancer metastasis (35–38).

The monocyte/macrophage cluster, which we identified, contains genes encoding proteins with variable intracellular localization and molecular function. Some of these proteins have been linked to cancer cell invasion, migration, and metastasis. For example, *Fyn* encodes a member of the Src family of protein kinases that are critical for macrophage migration (39), which promotes invasion and migration in breast cancer (40). *Enpp1* encodes an ecto-nucleotide pyrophosphatase/phosphodiesterase (ENPP), which is expressed in osteoclasts, and which promotes calcification of bone (41), and facilitates breast cancer metastasis to the bone (42). Both *Fyn* and *Enpp1* have been linked to resistance to tamoxifen treatment (43, 44). *Gab2*, which encodes a scaffolding adapter protein known to regulate EGF receptor signaling through ERK, is critical for the phagocytic function of macrophages (45), and was recently shown to promote breast cancer invasion and metastasis (46). *Glipr1*, which encodes glioma pathogenesis-related protein-1, has been linked to macrophage differentiation (47), and the invasive potential of melanoma cells (48).

Other genes within the monocyte/macrophage cluster have known roles in immune cells, but have been less studied in cancer. *Cd53* encodes a tetraspanin protein, which is among the most induced genes in macrophages exposed to lipopolysaccharide (49); *Fcer1g* encodes a high affinity receptor for IgE, which is critical for activation of mast cells and plays a key role in allergic inflammation (50). *Lrrk2*, which encodes leucine-rich repeat kinase 2, may play a role in monocyte maturation and variants of this gene is associated with an increased risk of both Parkinson’s and Crohn’s disease (51–53). *P2ry12* encodes a purinergic G protein-coupled receptor, which promotes macrophage chemotaxis and microglial activation (54, 55). *Relt* and *Nrip1*, which encode a member of the tumor necrosis factor receptor superfamily, and nuclear receptor-interacting protein-1, respectively, are both involved in activating NF-KappaB (56, 57). *Tbxas1*, *Dock8*, and *Wipf1*, which encode thromboxane A synthase 1, dedicator of cytokinesis 8, and WAS/WASL-interacting protein family member 1, respectively, are expressed in myeloid DCs and important for their polarization and migration (58–60). Some of the genes, such as *Fcgr2b*, which encodes the cell surface protein CD32; *Lsp1*, which encodes the lymphocyte-specific protein-1; and *Stab1*, which encodes the scavenger receptor Stabilin-1/CLEVER-1, are known to regulate monocyte/macrophage migration and endothelial cell interactions (61–63).

Interestingly, some of the genes in the monocyte/macrophage cluster have been linked to cellular stemness. In our microarray data, genes encoding cancer stem cell markers, such as CD44 and CD133, were not significantly upregulated. However, other genes known to regulate cellular stemness were induced. *Msi1*, which encodes Musashi1, an RNA binding protein, and *Wnt1*, are both master regulators of self-renewal in multiple stem cell populations (64, 65). *Sipa1* encodes a Rap1 GTPase-activating protein essential for proliferative control of hematopoietic progenitors (66). Upregulation of *Cdyl2* has been found in CD133-positive stem cells (67). Further studies are warranted to elucidate whether any of these genes contribute with stem cell properties to EMT cells.

In summary, our results indicate that tumor cells undergoing EMT in response to TGF-β1 do not simply turn into typical mesenchymal cells, such as fibroblasts. Rather, they acquire features of immune cells, in particular hematopoietic cells derived from myeloid precursors. The increased expression of genes in the monocyte/macrophage cluster in more aggressive breast cancer cell lines and tumors (EMT+, Basal B+, and ER−PR−) indicate that these genes might play roles in breast cancer metastasis. Clearly, further studies are needed to analyze the role of genes within the monocyte/macrophage cluster in providing cancer EMT cells with invasive and migratory properties. Such studies will include loss- and gain-of-function experiments to determine to the role of genes in providing EMT cells with the capacity to perform different steps of the metastatic process.

## Author Contributions

Study concept and design: Joel Johansson, Vedrana Tabor, Jonas Fuxe; acquisition and analysis of data: Joel Johansson, Vedrana Tabor, Anna Wikell, Jonas Fuxe; drafting of the manuscript: Joel Johansson, Vedrana Tabor, Jonas Fuxe; and funding: Jonas Fuxe; study supervision: Jonas Fuxe.

## Conflict of Interest Statement

The authors declare that the research was conducted in the absence of any commercial or financial relationships that could be construed as a potential conflict of interest.

## Supplementary Material

The Supplementary Material for this article can be found online at http://www.frontiersin.org/Journal/10.3389/fonc.2015.00003/abstract

## Footnotes

^1^http://david.abcc.ncifcrf.gov/

^2^http://biogps.org/

^3^http://www.ebi.ac.uk/
