# Supplementary material for: TGF-β1-Induced Epithelial–Mesenchymal Transition Promotes Monocyte/Macrophage Properties in Breast Cancer Cells
Source: Front Oncol. 2015 Jan 26;5:3. doi: 10.3389/fonc.2015.00003 (PMC4306317; doi:10.3389/fonc.2015.00003)
Supplement: Supplementary file 1 [file Data_Sheet_1.PDF]

Supplemental Information

TGF-β1-induced epithelial-mesenchymal transition promotes monocyte/macrophage properties in breast cancer cells

Joel Johansson, Vedrana Tabor, Anna Wikell, Sirpa Jalkanen and Jonas Fuxe

Inventory of Supplemental Information

Pages 2-4: **Table S1**

Page 5-7: **Table S2**

Page 8: **Table S3**

Page 9: **Figure S1**

Page 10: **Figure S2**

Page 11: **Figure S3**

Table S1. List of top 100 upregulated genes during TGF-β1-induced EMT in EpRAS cells

Fold	Chromosome	Public ID	Gene Symbol
15.9588	chr1	NM_027551	Klhl30
15.3873	chrX	NM_026838	Srpx2
13.0456	chr1	NM_011267	Rgs16
10.5218	chr3	NM_026335	Lce1h
10.182	chr5	NM_178730	Tmprss11f
9.5058	chr4	NM_011607	Tnc
9.3808	chr2	NM_013599	Mmp9
9.1422	chr2	NM_013822	Jag1
9.1364	chr5	NM_007786	Csn3
9.1235	chr6	NM_001080780	Ret
8.288	chr14	NM_177628	Fam167a
8.1363	chr15	NM_009865	Cdh10
8.0032	chr5	NM_027793	Amtn
7.5913	chr11	NM_016780	Itgb3
6.8203	chr6	NM_025331	Gng11
6.7676	chr13	NM_001081020	Adamts6
6.6353	chr11	NM_011340	Serpinf1
6.6326	chr15	NM_008216	Has2
6.075	chr8	NM_009868	Cdh5
6.0085	chr9	NM_011352	Sema7a
5.9972	chr19	NM_009985	Ctsw
5.6153	chr1	NM_172845	Adamts4
5.5327	chr11	NM_152818	Osbp2
5.4967	chr10	NM_009809	Casp14
5.275	chr13	NM_007796	Ctla2a
5.2136	chr9	NM_001172123	Rbms3
5.1904	chr14	NM_053105	Klhl1
5.1594	chr8	NM_008289	Hsd11b2
5.1111	chr15	NM_018865	Wisp1
4.9109	chr14	NM_138672	Stab1
4.8458	chr1	NM_010185	Fcer1g
4.7704	chr3	NM_177346	Gpr149
4.7208	chr11	NM_009616	Adam19
4.6424	chr2	NM_011824	Grem1
4.6057	chrX	NM_001024141	Nxf3
4.6051	chr7	NM_177469	Gpr123
4.4336	chr10	NM_001081127	Adamts14
4.3824	chr5	NM_146162	Tmem119

4.2672	chr18	NM_001039485	Fam38b
4.2173	chr4	NM_021436	Tmeff1
4.0186	chr7	NM_010248	Gab2
4.0173	chr5	NM_001177579	Gm10471
3.9935	chr17	NM_021332	Glp1r
3.9656	chr13	NM_008396	Itga2
3.9514	chr6	NM_001012477	Cxcl12
3.8717	chr9	NM_183103	Prss46
3.8706	chr13	NM_010104	Edn1
3.8405	chr3	NM_019978	Dclk1
3.8235	chr5	NM_001177579	Gm10471
3.7993	chr3	NM_029667	Lce1i
3.7971	chr9	NM_001003911	Adamts7
3.7808	chr14	NM_013492	Clu
3.713	chr8	NM_018827	Crlf1
3.7035	chr13	NM_011097	Pitx1
3.6808	chr7	NM_008350	Il11
3.6361	chr10	NM_028709	Btbd11
3.538	chr8	NM_172911	D8Ertd82e
3.4882	chr4	NM_001008791	Whrn
3.4503	chr5	NM_001134299	Gm10220
3.4498	chr16	NM_009655	Alcam
3.4489	chr18	NM_001039485	Fam38b
3.4297	chr2	NM_007389	Chrna1
3.4058	chr10	NM_010217	Ctgf
3.3806	chr19	NM_030703	Cpn1
3.3787	chr8	NM_028071	Cotl1
3.3585	chr3	NM_025413	Lce1g
3.3538	chr4	NM_029035	Spsb1
3.3382	chr15	NM_027496	Ankrd33b
3.3356	chr1	NM_008485	Lamc2
3.3012	chr6	NM_008970	Pthlh
3.2721	chr9	NM_028030	Rbpms2
3.2493	chr7	NM_019391	Lsp1
3.248	chr3	NM_010298	Glrb
3.2217	chr2	NM_022883	Lpin3
3.2119	chr18	NM_028341	Ttc39c
3.161	chr6	NM_013458	Add2
3.1505	chr12	NM_007483	Rhob
3.1426	chr7	NM_177448	Mogat2
3.1268	chr11	NM_029658	Fam101b
3.088	chr3	NM_133187	Fam198b

3.0725	chr13	NM_001177371	Dbn1
3.03	chr1	NM_173395	Fam132b
3.0176	chr9	NM_053178	Acsbg1
3.0088	chr2	NM_198029	Fermt1
2.9953	chr9	NM_008605	Mmp12
2.9775	chr10	NM_008813	Enpp1
2.9392	chr1	NM_001025602	Il1rl1
2.9368	chr4	NM_019753	Cdh17
2.9032	chr16	NM_021496	Pvrl3
2.8796	chr7	NM_001193305	Mical2
2.8632	chr15	NM_021279	Wnt1
2.8546	chr10	NM_021439	Chst11
2.8456	chr7	NM_009519	Wnt11
2.8084	chr1	NM_175460	Nmnat2
2.7951	chr9	NM_173781	Rab6b
2.7724	chr5	NM_008871	Serpine1
2.7648	chr1	NM_001114663	Plcl1
2.7645	chr2	NM_145535	Sdcbp2
2.732	chr19	NM_026619	Gsto2
2.7264	chr13	NM_007797	Ctla2b

Table S2. List of top 100 downregulated genes during TGF-β1-induced EMT in EpRAS cells

Fold	Chromosome	Public ID	Gene Symbol
-3.6436	chr2	NM_008008	Fgf7
-3.7191	chr6	NM_175314	Adamts9
-3.7244	chr6	NM_175314	Adamts9
-3.7444	chr2	NM_008714	Notch1
-3.7539	chr6	NM_153778	Atoh8
-3.8052	chr8	NM_008706	Nqo1
-3.8133	chr1	NM_011459	Serpinb8
-3.8161	chr6	NM_175314	Adamts9
-3.8371	chr7	NM_022420	Gprc5b
-3.8572	chr7	NM_009434	Phlda2
-3.8667	chr17	NM_011723	Xdh
-3.9181	chr7	NM_011464	Spint2
-3.9452	chr17	NM_010137	Epas1
-3.9469	chr7	NM_001164201	Lass3
-3.9667	chr11	NM_027984	Epn3
-3.9782	chr11	NM_008744	Ntn1
-4.0085	chr4	NM_030014	Hook1
-4.022	chr4	NM_172868	Palm2
-4.0529	chr7	NM_027897	Rhpn2
-4.0689	chr6	NM_022024	Gmfg
-4.0821	chr9	BC120577	Fam83b
-4.0831	chr4	NM_080555	Ppap2b
-4.1005	chr6	NM_198604	Plekhg6
-4.1006	chr16	NM_173379	Leprel1
-4.1346	chr9	NM_001040426	Thsd4
-4.2334	chr4	ENSMUST00000107600	Akap2
-4.2734	chr12	NM_001163136	Macc1
-4.2748	chr7	NM_028021	Myh14
-4.303	chr15	NM_033073	Krt7
-4.3437	chr16	NM_010143	Ephb3
-4.3836	chr4	NM_001017427	Rasef
-4.3861	chr18	NM_001146299	Sh3rf2
-4.4805	chr11	NM_008425	Kcnj2
-4.4894	chr11	NM_016958	Krt14
-4.6208	chr10	NM_008635	Mtap7
-4.7037	chrX	NM_010200	Fgf13
-4.8634	chr2	NM_016873	Wisp2
-4.8993	chr10	NM_026405	Rab32

-4.9729	chr15	NM_010738	Ly6a
-5.0119	chr12	NM_013464	Ahr
-5.039	chr6	NM_181344	C1rl
-5.0445	chr7	NM_023908	Slco3a1
-5.0908	chr19	NM_181404	Kank1
-5.1425	chr16	NM_011782	Adamts5
-5.2577	chr14	NM_007554	Bmp4
-5.3148	chr8	NM_008630	Mt2
-5.3193	chr9	NM_007962	Mpzl2
-5.374	chr5	NM_008009	Fgfbp1
-5.4317	chr13	NM_001038602	Marveld2
-5.6666	chr11	NM_011333	Ccl2
-5.7867	chr2	NM_010495	Id1
-5.805	chr6	NM_001113356	C1rb
-5.9721	chr1	NM_009061	Rgs2
-6.1705	chr18	NM_001001488	Atp8b1
-6.4023	chr13	NM_024272	Ssbp2
-6.4282	chr13	NM_177809	Slc25a48
-6.5417	chr4	NM_008321	Id3
-6.6183	chr10	NM_011595	Timp3
-6.6649	chr10	NM_172393	Aim1
-6.6986	chr1	NM_144796	Susd4
-6.727	chr1	NM_001003948	Pid1
-6.7377	chr6	NM_017379	Tuba8
-6.8462	chr13	NM_008046	Fst
-7.0755	chr6	NM_011375	St3gal5
-7.1081	chr10	NM_009933	Col6a1
-7.1249	chr15	NM_009640	Angpt1
-7.298	chr6	NM_007472	Aqp1
-7.3041	chr14	NM_053115	Acox2
-7.4229	chr2	NM_001159564	Itgb6
-7.4386	chr7	NM_017405	Lsr
-7.5349	chr15	NM_001164627	Arhgap8
-7.9053	chr2	NM_053195	Slc24a3
-8.0256	chr7	NM_017465	Sult2b1
-8.1023	chr2	NM_015736	Galnt3
-8.5537	chr14	NM_201529	Lmo7
-9.066	chr15	NM_026496	Grhl2
-9.5488	chr13	NM_008756	Ocln
-9.6006	chr11	NM_175263	Notum
-9.89	chr7	NM_133712	Klk10
-10.7227	chr1	NM_016851	Irf6

-10.7295	chr6	NM_145826	Il17re
-10.7998	chr6	NM_198884	B4galnt3
-11.0256	chr5	NM_001161548	Tmem184a
-11.1407	chr9	NM_001110300	Ap1m2
-11.5339	chr2	NM_011414	Slpi
-12.8803	chr17	NM_009994	Cyp1b1
-14.7566	chr16	NM_008909	Ppl
-14.8874	chr11	NM_016887	Cldn7
-14.9431	chr17	NM_008532	Epcam
-16.2461	chr9	NM_011176	St14
-16.4002	chr12	NM_010496	Id2
-16.4373	chr4	NM_025452	Tmem54
-18.2239	chr8	NM_008430	Kcnk1
-19.0288	chr2	NM_010762	Mal
-19.6451	chr15	NM_145469	Nipal2
-31.9854	chr1	NM_009257	Serpinb5
-35.9066	chr8	NM_009864	Cdh1
-42.5296	chr4	NM_194055	Esrp1
-52.6139	chr6	NM_020047	Tacstd2
-59.6688	chr3	NM_016899	Rab25

Table S3: Clustering of genes upregulated or downregulated according to cellular function.

Term	Genes
**Upregulated**	
GO:0048762~mesenchymal cell differentiation	Acvr1, Edn1, Rdh10, Ednra, Zeb2, Ret, Efnb1
GO:0016477~cell migration	Vegfa, Tgfb2, Ppard, Pvr, Cxcl12, AI836758, Nav1, Hbegf, Ret, Pdgfb, Fcer1g, Fyn, Acvr1, Nr4a2, Tns1, Gab2, Zeb2, Efnb1
GO:0006928~cell motion	Tgfb2, Vegfa, Ephb1, Ppard, AI836758, Hbegf, Ret, Fcer1g, Fyn, Robo1, Acvr1, Gab2, Zeb2, Alcam, Etv4, Pvr, Cxcl12, Nav1, Pdgfb, Atp1a3, Nr4a2, Tns1, Bmp7, Efnb
GO:0022610~biological adhesion	Tgfb2, Lamb3, Pvrl1, Itga7, Stab1, Igsf5, Ppard, Itga5, Tnc, Pvrl3, Col16a1, Ncam1, Col7a1, Ret, Col5a3, Cdh23, Rhob, Alcam, Pvr, Pcdhb18, Amtn, Adam12, Lamc2, Cdh5, Wisp1, Zyx, Itga2, Cdh10, 5830467P10Rik, Plekhc1, Itgb3, Cdh17, Mcam, Pkp2, Cyr61
GO:0008284~positive regulation of cell proliferation	Vegfa, Tgfb2, Ppard, Pdgfa, Vegfc, Hbegf, Pdgfb, Edn1, Vash2, Pthlh, Clu, Il11
GO:0007010~cytoskeleton organization	Nuak2, Dbn1, Tnnt2, Pdgfa, Tmsb4x, Mtap1s, Nav1, Tsr2, Pdgfb, Fhod3, 2310039E09Rik, Lsp1, Mtap1b, Pstpip2, Pdlim7, Tagln
**Downregulated**	
GO:0030855~epithelial cell differentiation	Krt14, Ppl, Notch1, A130010J15Rik, Crb3, Esr1, Id1, Id3, Fzd1
GO:0060429~epithelium development	Krt14, Notch1, A130010J15Rik, Crb3, Serpinb5, Id1, Nrp1, Eya1, Dlc1, Ppl, Bmp4, Celsr1, Esr1, Cobl, Id3, Fzd1,
GO:0007155~cell adhesion	Gpnmb, Cdon, Wisp2, Pkp3, Ctnnal1, Nrp1, Cldn7, Col6a1, Mpzl3, Ptprf, Pcdh9, Dlc1, Perp, Antxr1, Cdh1, Ly9, Mpzl2, Amigo2, Cadm1, Celsr1, Itgb6, Kitl, Aebp1
GO:0010942~positive regulation of cell death	Tgfb2, Ppp1r13b, Ercc6, Mmp9, Casp14, Arhgef7, Bmp7

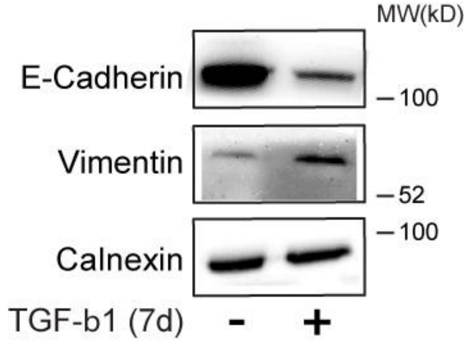

Figure S1

Figure S1. Western blot analysis on cells treated 7 days with 2ng/ml TGF-β1. Immunoblotting showing decreased levels of E-Cadherin and increased levels of Vimentin after 7 days of TGF-β1 exposure. Calnexin was used as a loading control.

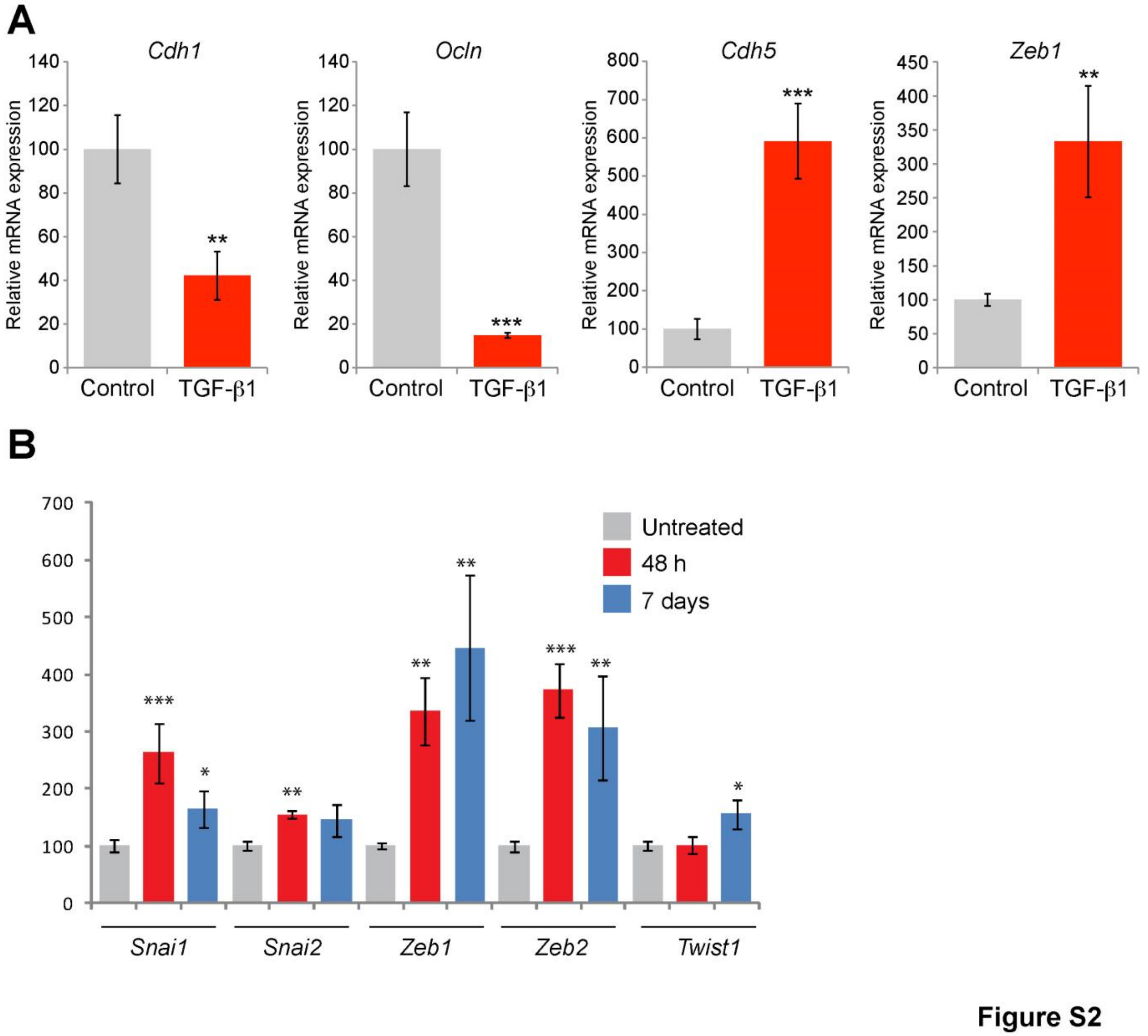

Figure S2. (A) Validation of microarray data by qPCR. Results from qPCR analysis showing changes in gene expression of two epithelial markers: *Cdh1*, *Ocln*; and two mesenchymal markers: *Cdh5*, *Zeb1*. (B) Results from qPCR analysis showing changes in gene expression of master regulators of EMT at 48 h and 7 d after TGF-β1 treatment. * = P≤0.05; ** = P≤0.01; *** = P≤0.001

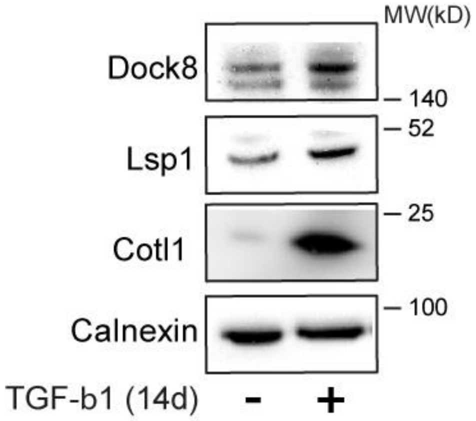

Figure S3

Figure S3. Validation of monocyte/macrophage profile by Western Blot. Results from western blot analysis showing changes in protein expression of Dock8, Lsp1 and Cotl1, three genes belonging to the monocyte/macrophage profile, after 14 days of TGF-β1 treatment. Calnexin was used as a loading control.
